# Artificial Loading of ASC Specks with Cytosolic Antigens

**DOI:** 10.1371/journal.pone.0134912

**Published:** 2015-08-10

**Authors:** Ali Can Sahillioğlu, Nesrin Özören

**Affiliations:** 1 Apoptosis and Cancer Immunology Laboratory (AKiL), Department of Molecular Biology and Genetics, Bogazici University, Istanbul, Turkey; 2 Life Sciences and Technologies Research Center, Bogazici University, Istanbul, Turkey; University of the Pacific, UNITED STATES

## Abstract

Inflammasome complexes form upon interaction of Nod Like Receptor (NLR) proteins with pathogen associated molecular patterns (PAPMS) inside the cytosol. Stimulation of a subset of inflammasome receptors including NLRP3, NLRC4 and AIM2 triggers formation of the micrometer-sized spherical supramolecular complex called the ASC speck. The ASC speck is thought to be the platform of inflammasome activity, but the reason why a supramolecular complex is preferred against oligomeric platforms remains elusive. We observed that a set of cytosolic proteins, including the model antigen ovalbumin, tend to co-aggregate on the ASC speck. We suggest that co-aggregation of antigenic proteins on the ASC speck during intracellular infection might be instrumental in antigen presentation.

## Introduction

Inflammasomes are cytosolic complexes, involved in sensing PAMPs from a broad range of viral and bacterial pathogens [1–3]. Inflammasome complexes contain receptors (such as NLRP3, NLRC4 and AIM2) that oligomerize upon activation [4]. The oligomerized inflammasome receptors recruit the apoptosis-associated speck-like protein containing a CARD (ASC) which interacts with inactive zymogen procaspase-1. Thereby, procaspase-1 proteins that are brought into a closed proximity cleave each other. The processed mature caspase-1 is capable of hydrolyzing prointerleukin-1beta and prointerleukin-18, the mature forms of which are subsequently secreted [4].

It has been shown that upon activation, inflammasome components form a micrometer-sized supramolecular structure called the ASC specks/ASC foci/pyroptosome near the nucleus [5–7]. Recently, prion-like polymeric structure of the ASC speck has been unraveled [8–10]. This structure has been shown to have a role in the induced proximity mediated procaspase-1 self-activation [6]. Although the ASC speck is thought to be an enzymatic platform for inflammasome activity, it is comparably much bigger than oligomeric DISC and apoptosome structures which regulate caspase-8 and caspase-9 activation, respectively [11]. The ASC speck appears to be an enzymatic platform with unorthodox physical properties which raises the question, whether aggresome-like properties of the ASC speck has any advantage over oligomeric activation platforms.

We report here that the ASC speck has an intrinsic properly to co-aggregate cytosolic proteins. The co-aggregation of cytosolic proteins on the ASC speck appears to be based on non-specific hydrophobic interactions. Protein aggregation in the form of dendritic cell aggresome-like induced structures (DALIS) was shown to be important in antigen presentation [12–14]. Given the inflammasome-dependent and-independent roles of ASC protein in antigen presentation [15–19], we hypothesized that the aggresome-like properties of the ASC speck might be the underlying reason of the ASC speck formation.

The inflammasomes are functional in professional antigen presenting cells such as dendritic cells (DCs) and macrophages as well as in other cell types that are frequently in contact with pathogens, such as keratinocytes and neutrophils [20]. Alum, a widely used adjuvant in human vaccines, has been shown to exert its adjuvant activity via stimulation of NLRP3 inflammasome in an ASC dependent manner [15,16]. Furthermore, ASC protein has also been implicated in antigen presentation via inflammasome independent mechanisms [17–19]. Yet, the ability of ASC specks to co-aggregate cytosolic proteins has not been shown before.

In this paper, we discuss three alternative scenarios that antigen co-aggregation on the ASC speck might contribute to antigen presentation depending on further events following inflammasome activation: cytosolic localization [5] and autophagic clearance [21] of ASC specks and pyroptotic cell death [22]. Our previous findings suggest that the ASC speck is a highly organized structure, yet the co-aggregation of cytosolic proteins on the ASC speck is based on non-specific protein aggregation [10]. In that sense, we propose the ASC speck as a novel scaffold which is formed at the right time at the right place during intracellular infection and might be important in antigen presentation.

## Results and Discussion

**Fig 1:**
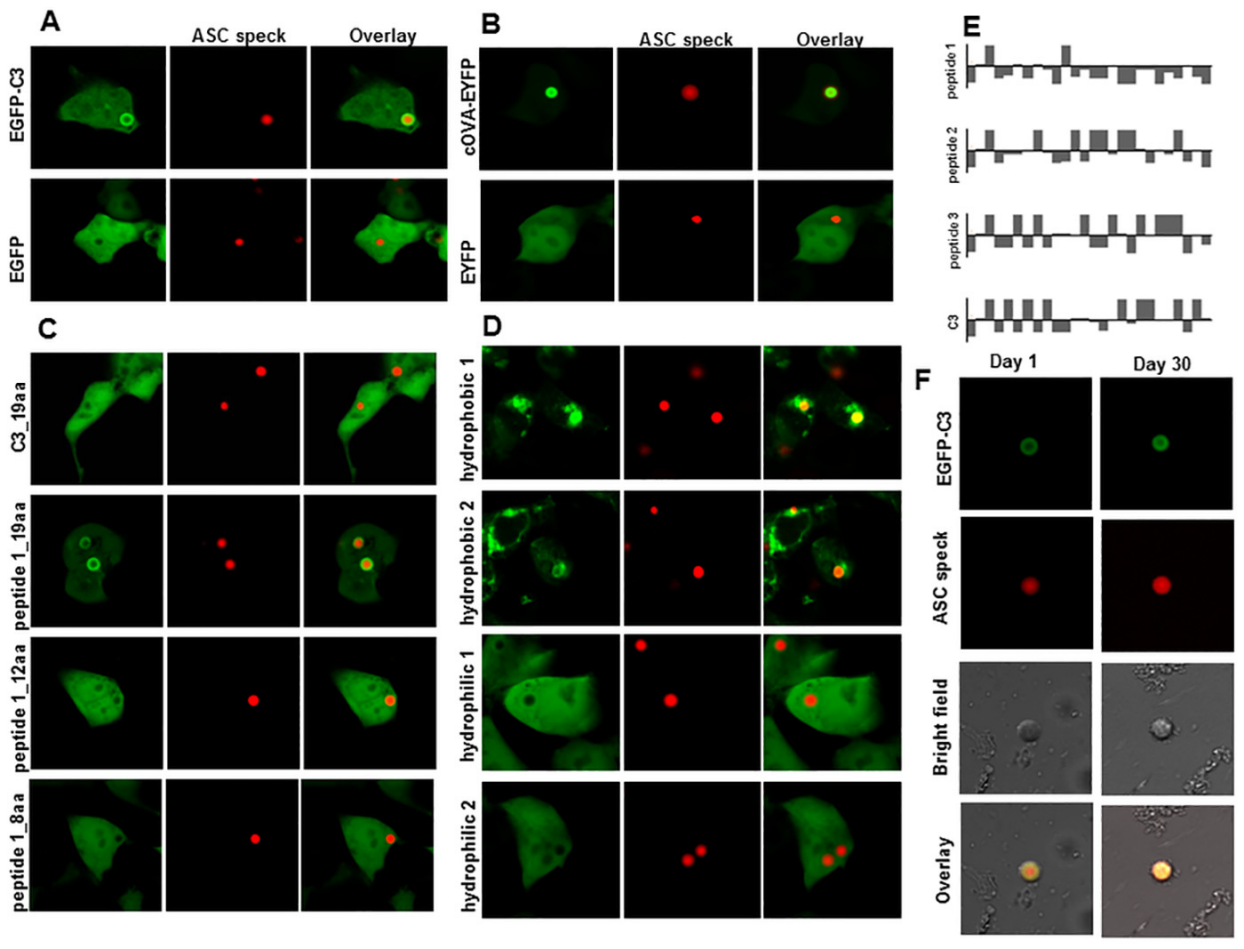
ASC specks stably co-aggregate some but not all cytosolic proteins. Fluorescently tagged (mCherry) ASC protein was co-expressed with a set of EGFP-tagged constructs in HEK293T cells. Representative constructs: (A) EGFP-C3 and EGFP alone (B) cOVA-EYFP (1-48aa secretion signal deleted) and EYFP alone were co-expressed with mCherry-ASC. See S1 Fig for other constructs tested. (C) Shorter versions of C3 and peptide 1 were cloned to C-terminus of EGFP. Only EGFP-peptide 1_19aa co-aggregated on ASC specks, but EGFP-C3_19aa, -peptide 1_12aa and-peptide 1_8aa did not. (D) 2 hydrophobic and 2 hydrophilic randomly generated peptide encoding sequences were cloned to C-terminus of EGFP. EGFP-hydrophobic peptides but not EGFP-hydrophilic peptides co-aggregated on ASC specks. Results are representative of two independent experiments. (E) Hydropathy plots of peptides co-aggregating (peptide 1 and C3) or not co-aggregating (peptide 2 and 3) on ASC specks when fused with EGFP and co-expressed with mCherry-ASC construct. Y-axis: Hydrophobicity values according to [38]. X-axis: Amino acid position. Columns above the x-axis correspond to hydrophilic peptides and below the x-axis correspond to hydrophobic peptides. (F) EGFP-C3 co-aggregated ASC specks were extracted from HEK293T cells. Samples were imaged right after extraction and after incubation at 37°C for 30 days in PBS. Results are representative of at least two independent experiments. Scale bar: 10 μm.

ASC proteins form a perinuclear aggregate called the ASC speck upon inflammasome activation S1 Fig. Similar perinuclear aggregates spontaneously formed upon overexpression of mCherry-tagged ASC protein in HEK293T cells (Fig 1). Spontaneous ASC speck formation upon overexpression of ASC has been described before, which is thought to be due to excess of critical concentration of ASC protein so that the ASC speck forms without the need for activation by inflammasome receptors [23, 24].

Interestingly, when ASC was co-expressed with EGFP protein fused to a panel short peptides, a subset of EGFP-peptide fusion proteins co-aggregated on the ASC speck, but EGFP alone did not (Fig 1A). Similarly, ASC speck co-aggregated the model antigen ovalbumin labeled with EYFP, but not EYFP alone (Fig 1B). The diversity of cytosolic proteins which could co-aggregate on the ASC speck suggests that the co-aggregation is non-specific.

We found our initial observation of cytosolic protein co-aggregation on the ASC speck interesting, as it bears the potential to be important in antigen presentation and help to explain why ASC proteins form perinuclear aggregates upon inflammasome activation. Thereby, we carried out a series of experiments to characterize this co-aggregation.

The first construct observed to co-aggregate on the ASC speck is EGFP-C3 (Fig 1A). The protein product is encoded by the commercial plasmid pEGFP-C3, which encodes EGFP protein with a C-terminal multiple cloning site encoding 26 amino acids long peptide. Later, 3 randomly chosen peptides of the same length from the ampicillin resistance gene were cloned at the C-terminus of the EGFP. EGFP-peptide 1 co-aggregated on the ASC speck whereas EGFP-peptide 2 and-peptide 3 did not (Fig 1C and 1D). A shorter version of peptide 1 (peptide 1_19aa) fused to EGFP co-aggregated on the ASC speck but other shorter peptides, namely peptide 1_12aa, peptide 1_8aa and C3_19aa failed to co-aggregate (Fig 1C and 1D). Peptide 1 clearly has a hydrophobic amino acid sequence although hydrophobicity of C3 is not obvious (Fig 1C and 1D). We randomly generated and cloned 2 hydrophobic and 2 hydrophilic peptides at the C-terminal of EGFP and observed that EGFP-hydrophobic peptides co-aggregated on the ASC speck, but EGFP-hydrophilic peptides did not (Fig 1C and 1D). Non-specific hydrophobic interactions appear to contribute to co-aggregation of different EGFP-peptide constructs on the ASC speck.

Next, we extracted EGFP-C3 co-aggregated ASC specks from HEK293T cells. Extracts survived repeated sonication cycles and extended incubation at 37°C for 30 days, which indicates the mechanical stability of the structure (Fig 1F). Also, the presence of co-localization after extraction suggests that EGFP-C3 is stably co-aggregated on the ASC specks rather than transiently co-existing at a membrane enclosed organelle such as autophagosome.

To rule out the possibility of any interaction between fluorescent labels which can be accounted for the co-aggregation of cytosolic proteins at ASC specks, we expressed cOVA-EYFP alone or co-expressed with unlabelled ASC, NLRP3, procaspase-1 in HEK293T cells (S2 Fig). In the absence of inflammasome components, cOVA-EYFP showed a diffused cytosolic expression pattern. However, when cOVA-EYFP is co-expressed with inflammasome components, speck structures were observed at a high frequency. Speck structures were not observed when EYFP is expressed alone or co-expressed with inflammasome components.

**Fig 2:**
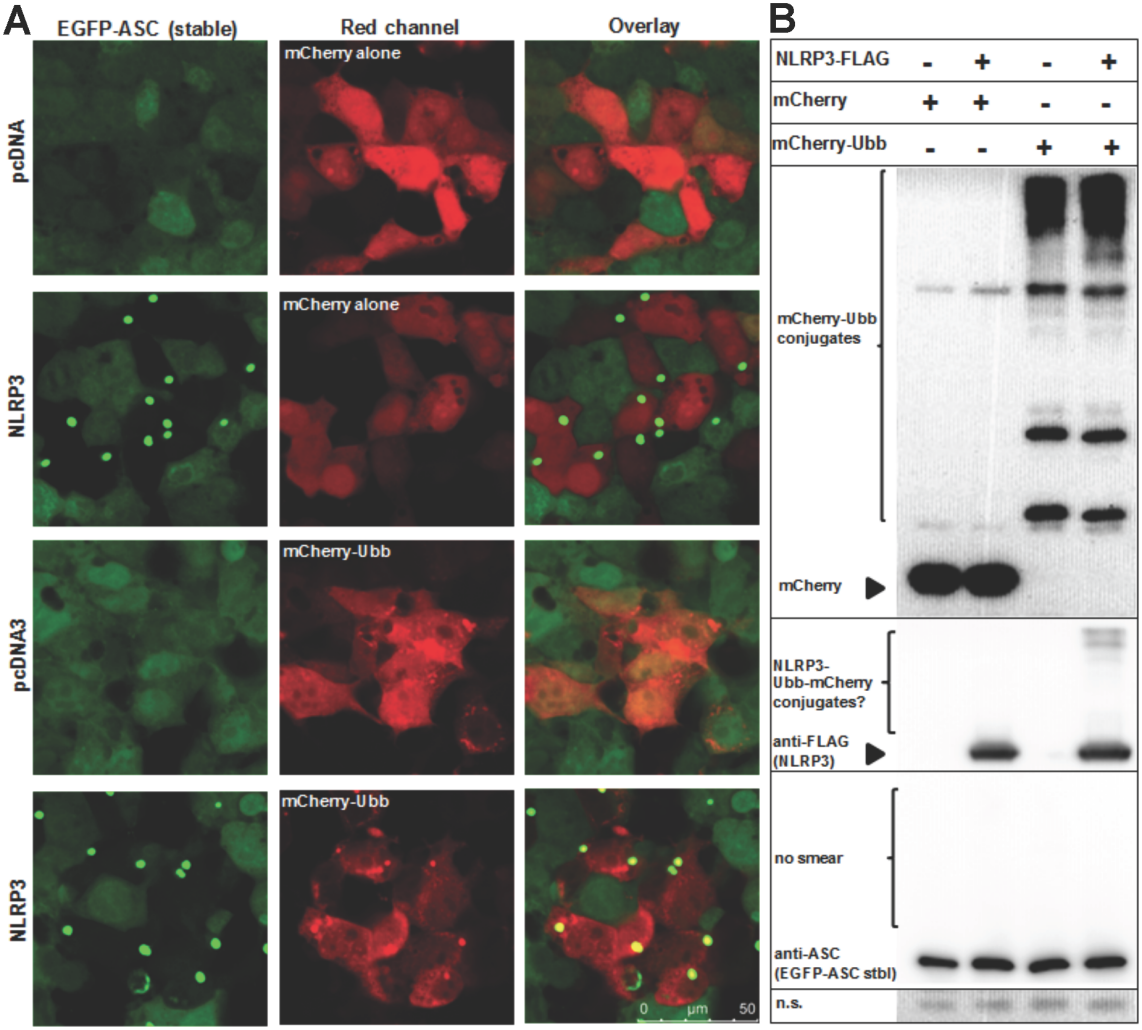
Ubiquitin B is co-localized on NLRP3-induced ASC specks. HEK293T cells were transduced with lentiviral particles to stably express EGFP-ASC fusion protein. (A) Confocal micrograph images of cells that were either transfected with empty (pcDNA3) or NLRP3-encoding plasmids to induce ASC speck formation. Cells were also co-transfected with either mCherry alone (control) or mCherry-UBB (ubiquitin B). Co-localization of ASC specks with mCherry-UBB but not with mCherry alone was observed. (B) Western blotting analysis of samples in (A). High molecular weight bands in mCherry-UBB expressing cell lysates were detected, which are thought to be conjugates formed by UBB. Similar bands were also observed when NLRP3 and mCherry-UBB were co-transfected, suggesting a band shift due to conjugation of NLRP3 with mCherry-UBB. No high molecular smear was observed for ASC. N.s.(non-specific): loading control. Results are representative of two independent experiments. Scale bar: 25 μm.

Ubiquitination of inflammasome components, but not ASC, and the presence of ubiquitin on the ASC speck have been shown before [18]. We checked the presence of ubiquitin B (UBB) on the NLRP3-induced ASC specks. HEK293T cells that stably expressing ASC protein at physiological levels were transfected with either empty or NLRP3-encoding plasmids. Around 50% of cells produced ASC specks when transfected with NLRP3-encoding plasmid, whereas less than 1% of cells spontaneous formed ASC specks when transfected with empty plasmid. We observed co-localization of UBB on ASC specks in almost all ASC speck forming and mCherry-UBB (+) cells, whereas mCherry alone did not co-localize on the ASC speck (Fig 2).

**Fig 3:**
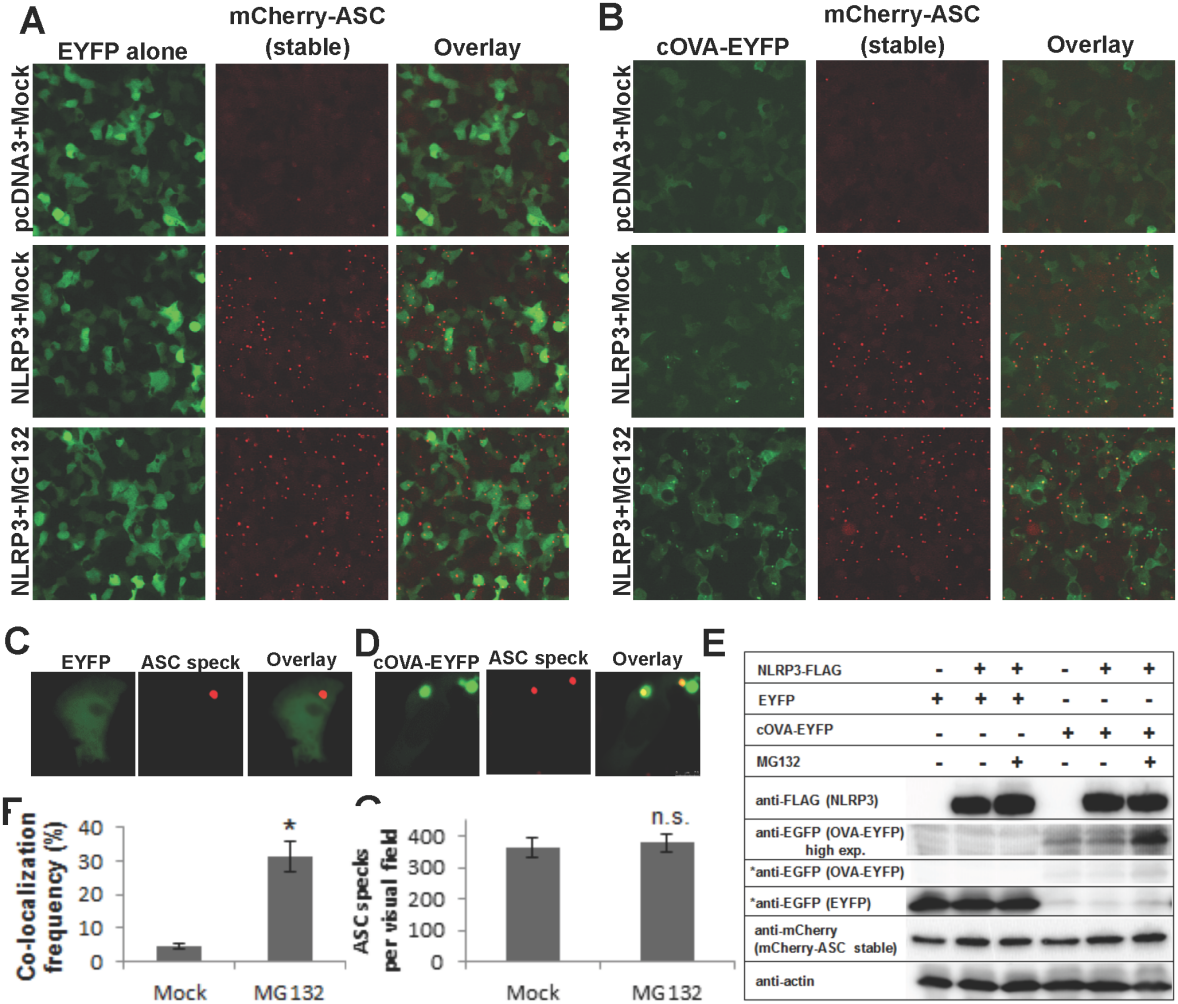
Co-localization frequency of ovalbumin on NLRP3-induced ASC specks is increased by proteasomal inhibition. HEK293T cells were transduced with lentiviral particles to stably express mCherry-ASC fusion protein. Inverted fluorescent microscopy images of cells, transfected either with empty (pcDNA3) or NLRP3-encoding plasmids, to induce ASC speck formation. Cells were also co-transfected with either (A) EYFP alone (control) or (B) cOVA-EYFP Scale bar (A) and (B): 100 μm. 24 h after transfection, cells were treated with either mock or 10 μM MG132 for 6 h. Exposure time of EYFP alone images is kept 4x shorter than cOVA-EYFP to avoid saturation of pixels due to fluorescence intensity differences. Close-up confocal micrographs of (C) EYFP or (D) cOVA-EYFP expressing NLRP3-induced ASC speck forming cells. Scale bar (C) and (D): 10 μm (E) Western blotting analyses of samples in A-B. EYFP and cOVA-EYFP were exposed equally (marked with asterix). cOVA-EYFP was also exposed longer due to low intensity of the bands. (F) Co-localization frequency of ovalbumin on NLRP3-induced ASC specks either in the absence or presence of MG132 (p < 0.0001, n = 4). (G) NLRP3-induced ASC specks per visual field either in the absence or presence of MG132 (not significant, p = 0.48, n = 4). Results are representative of two independent experiments.

To find out whether cytosolic protein co-aggregation is present at physiological ASC concentrations, we carried out a similar experiment using HEK293T cells stably expressing ASC. We observed 4.63 ± 0.83% co-localization frequency of cOVA-EYFP on the NLRP3-induced ASC specks, whereas EYFP alone showed no co-localization (Fig 3A–3D). Moreover, co-localization frequency of ovalbumin on ASC specks increased to 31.3 ± 4.4% upon proteasomal inhibition by MG132 (Fig 3E). MG132 treatment increased ovalbumin protein level as observed by Western blotting and fluorescence microscopy (Fig 3B and 3E). No change was observed in ASC speck formation frequency or in protein levels of inflammasome components (Fig 3E and 3G). It appears that MG132 treatment increases cellular levels of ovalbumin, which in turn increases its co-localization frequency on the ASC speck. This finding is important to show that the ASC speck is capable of co-aggregating ubiquitinated proteins. Aggresome and aggresome-like structures (DALIS and JUNQ) contain ubiquitinated proteins and their formation is increased by proteasomal inhibition [14, 25, 26]. The ASC speck formation is not affected by proteasomal inhibition, yet co-aggregation of ovalbumin is favored by MG132. This result indicates that the ASC speck formation and co-aggregation of ovalbumin on the ASC speck are two different processes. The former depends on specific interactions between ASC proteins as we have shown previously, and the latter depends on protein aggregation.

Co-aggregation of cytosolic proteins on the ASC speck has potential implications in antigen presentation. Previous studies indicate that protein aggregation in form of DALIS functions as a cytosolic storage and thereby prolongs antigen presentation [13]. It is possible that the ASC speck might modulate antigen stability similar to DALIS. The second scenario involves autophagy, as previous studies showed the autophagic clearance of the ASC speck [21]. While cytoplasmic proteins are mostly presented via MHC class I molecule, cytoplasmic proteins that enter autophagic pathway are presented via MHC class II in a process called cross-presentation [27]. Again, DALIS was shown to contribute cross-presentation via autophagic pathway [12]. Thereby, cytosolic protein co-aggregation on the ASC speck is likely to contribute to cross-presentation. Strikingly, inflammasome activation was reported to be coupled by secretion of inflammasome components and MHC class II containing exosomes [28, 29]. While the authors suggested that MHC-II mediated release of IL-1β and inflammasome components might explain unconventional (ER-Golgi independent) secretion of IL-1β, such an autophagy-MHC-II involving pathway might be instrumental in cross-presentation of antigens co-aggregated on the ASC speck.

**Fig 4:**
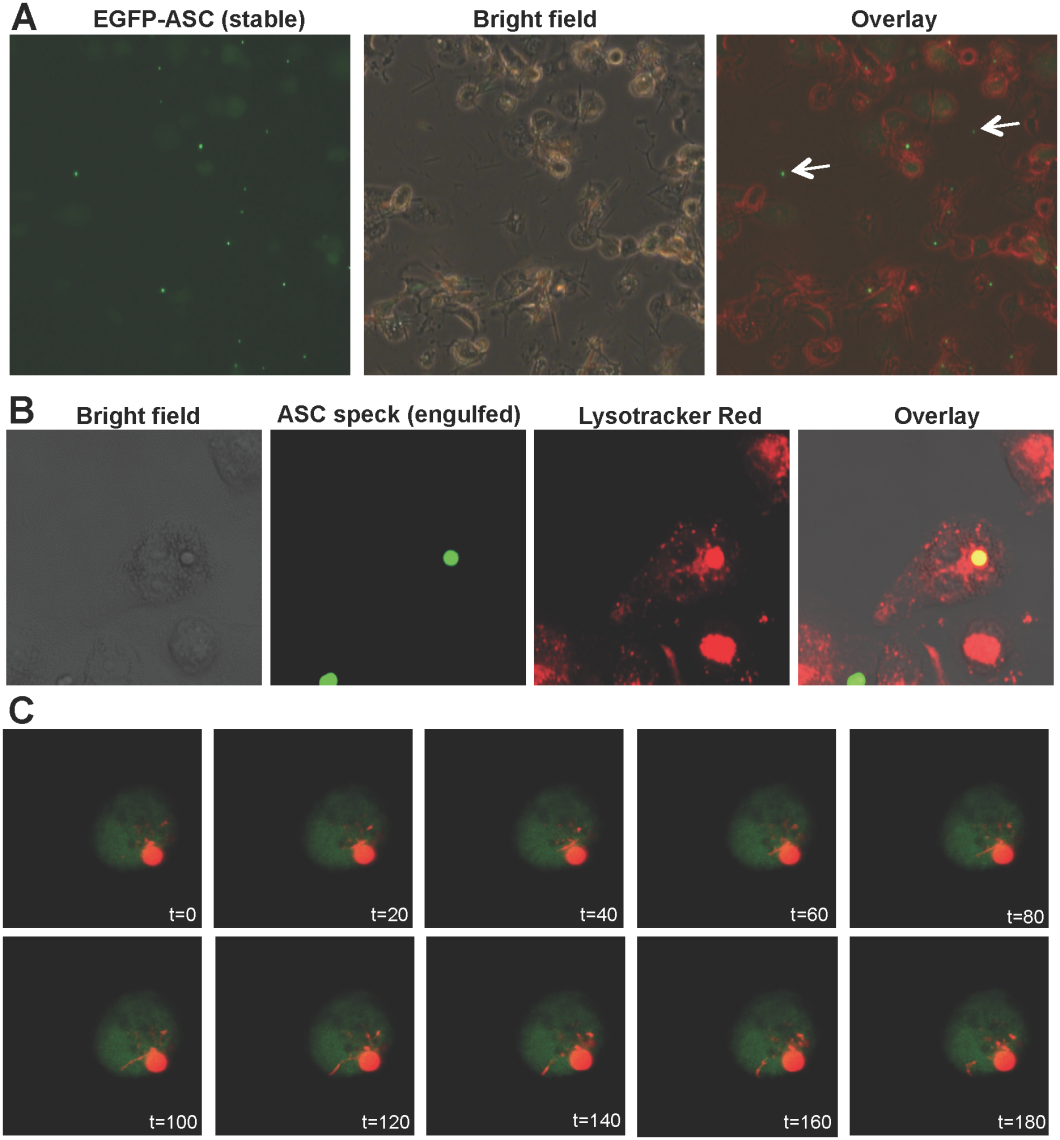
Extracellular ASC specks were released from THP-1 macrophages treated with MSU crystals, and can be engulfed and processed by another macrophage. (A) Human monocytic THP-1 cells were transduced with lentiviral particles to stably express EGFP-ASC fusion protein. THP-1 cells were differentiated with PMA. Differentiated cells were treated with 150 μg/ml MSU crystals for 24 h. ASC speck were observed in the extracellular space (marked with arrows). Bright field is converted into red channel in the overlay image for better visual seperation. Scale bar (A): 100 μm. (B) EGFP-tagged ASC specks were purified from HEK293T cells and incubated with PMA-differentiated THP-1 cells for 3 h. Presence of engulfed ASC speck in an acidic organelle in THP-1 macrophage was demonstrated by Lysotracker Red staining. (C) Time-lapse imaging of PMA-differentiated stably EGFP-ASC expressing THP-1 macrophages that engulfed extracellular mCherry-tagged ASC specks. Tubular vesicles trafficking from the phagolysosome which contained engulfed ASC speck were observed. Cytosolic stable EGFP-ASC expression (diffused green signal) and engulfed mCherry-ASC speck were observed as two distinct compartments within the same cell. Time-lapse imaging was carried out using an in-house produced growth chamber. Results are representative of at least two independent experiments. Scale bar (B) and (C): 25 μm.

While a series of studies show that inflammasome activation is accompanied by pyroptotic cell death, it is evident that not every activated cell dies upon inflammasome activation. The ASC speck is also referred as “pyroptosome” and it is involved in caspase-1 dependent cell death, although contradictory studies are present [5, 6]. Pyroptotic cell death is characterized by release of cytosolic content to extracellular environment [22]. Indeed, we have observed extracellular ASC specks upon prolonged incubation of PMA-differentiated THP-1 macrophages with MSU crystals (Fig 4A). Furthermore, we showed that THP-1 cells are able to engulf purified extracellular ASC specks. Engulfed ASC specks are present in an acidic organelle, most likely the phagolysosome (Fig 4B). Time-lapse imaging revealed that engulfed ASC specks are processed, as tubular vesicles pinching off from the phagolysosome were observed (Fig 4C). Previous studies showed that these type of tubular vesicles are responsible for MHC-II dependent presentation of extracellular antigens [30–32]. When THP-1 cells are imaged by time-lapse confocal microscopy without a growth chamber over >30 minutes, cells died by undergoing membrane blebbing. Under these conditions, the membrane enclosing engulfed ASC speck became apparent. We observed that the space between phagolysosomal membrane and the intact engulfed ASC speck filled with degradation products of the ASC speck, with weaker fluorescence intensity. The gradual degradation of engulfed ASC speck in the phagolysosome is similar to controlled release applications achieved by particulate antigen delivery vehicles [33]. Briefly, antigens are loaded into nano- and micrometer sized particles in order to enhance engulfment of the antigen by increasing its size, prolong antigen stability by limiting enzymatic degradation, and thereby increase the duration of antigen presentation in vaccination studies. These particles are often decorated with TLR-ligand immunomodulators [33]. Interestingly, upon activation of AIM2 inflammasome, cytosolic DNA coming from the pathogen localizes on the ASC speck [7]. These pathogenic, mostly unmethylated DNA and its analog (CpG) is the ligand of TLR9 [33]. Similary, flagellin activates both NLRC4-NAIP5 and TLR5 pathways and physically interacts with NLRC4-NAIP5 complex [34,35]. However for our knowledge, localization of flagellin on the ASC speck has not been reported. Two recent studies showed that extracellular ASC specks are involved in propagation of inflammation [36,37]. Collectively, extracellular ASC specks have remarkable similarities with particulate vaccines.

In conclusion, we showed that the ASC speck has an intrinsic ability to co-aggregate cytosolic proteins. Our data shows that ubiquitinated proteins, proteins with hydrophobic patches co-aggregate on the ASC speck. Antigen co-aggregation on the ASC speck has potential implications in antigen presentation, which might be employed in rational vaccine design.

## Methods

### Plasmids

pEGFP-hASC and pmCherry-hASC plasmids were cloned by subcloning human ASC cDNA derived from pcDNA3-hASC plasmid into pEGFP-C3 (Clontech, USA) and pmCherry-C3.1 (in-house produced) vectors between HindIII and EcoRI sites. pcOVA-EYFP plasmid was cloned by subcloning of cytoplasmic ovalbumin cDNA (1-48aa secretion signal deleted) from pCI-neo-sOVA plasmid (Addgene plasmid 25098, kindly provided by Prof. Pedro Lowenstein, Cedars-Sinai Medical Center) into pEYFP-N1 vector (Clontech, USA) between NheI-BglII sites. Ubiquitin B cDNA was subcloned from pCGN-HA-Ubiquitin plasmid into pmCherry-C3.1 to obtain pmCherry-UBB. DNA sequences encoding short peptides were cloned between BglII and EcoRI sites of pEGFP-C3 vector (S1 Table). pEGFP alone and pmCherry alone plasmids have the same vector backbone as pEGFP-C3 except they lack the multiple cloning site. EGFP-ASC encoding DNA from pEGFP-hASC and mCherry-ASC encoding DNA from pmCherry-hASC were subcloned into pLenti-Ef1a vector to yield lentiviral plasmids pLenti-Ef1a-EGFP-ASC and pLenti-Ef1a-mCherry-ASC. These constructs were used together with packaging plasmids pCMVdeltaR8.74 and pMD2.G plasmids for virus production. pCMVdeltaR8.74 and pMD2.G were kindly provided by Prof. Karl Deisseroth (Stanford University). pcDNA3-NLRP3-FLAG, pcDNA3-hASC, pcDNA3-procaspase-1-FLAG, pCGN-HA-Ubiquitin plasmids were kindly provided by Prof. Gabriel Nunez (University of Michigan).

### Transfection

HEK29T cells were transfected with standard calcium phosphate transfection method.

### Stable lines

HEK293T cell lines stably expressing either EGFP-ASC or mCherry-ASC and THP-1 cell line stably expressing EGFP-ASC were created by lentiviral transduction, following selection of single cells in 96-well plates.

### PMA differentiation

Monocytic THP-1 cells were differentiated into macrophages by 0.5 μM phorbol 12-myristate 13-acetate (PMA) treatment for 3 h, followed by 24 h incubation.

### Imaging

Cells were imaged with TCSSP5II confocal microscope (Leica, Germany) and Axio Observer inverted fluorescent microscope (Zeiss, Germany). Nuclei and lysosomes were stained with DAPI and Lysotracker Red according to manufacturer’s instructions, respectively. (Invitrogen, USA).

### Purification of ASC specks

HEK293T cells were transfected with ASC encoding plasmids by calcium phosphate method. 24 h after transfection, cells were lysed by sonication. ASC specks were sepearated from soluble proteins and enriched by repeated low speed centrifugation cycles at 200 g.

### Antibodies

Anti-FLAG (2368S, CST, USA), anti-Caspase-1 (sc-515, Santa Cruz), anti-EGFP (Vatoz, Turkey), anti-ASC (kindly provided by Dr Masumoto, Jichi Medical University), anti-mCherry (kindly provided by Dr Arzu Celik, Bogazici University) were used for Western blotting.

### Cell culture

HEK293(F)T cells were kindly provided by Prof. Maria Soengas (CNIO) and maintained in DMEM containing 10% FBS. THP-1 cells were kindly provied by Prof. Ahmet Gül (Istanbul University) and maintained in RPMI containing 10% FBS.

## Supporting Information

S1 Fig Co-aggregation of EGFP-peptide constructs on ASC specks. Three randomly chosen 26 amino acid long peptide encoding sequences from ampicillin resistance gene were cloned to C-terminus of EGFP. When co-expressed with mCherry-tagged ASC in HEK293T cells, only EGFP-peptide 1 co-aggregated on ASC speck, but EGFP-peptide 2 and-peptide 3 did not. EGFP-hydrophobic peptides but not EGFP-hydrophilic peptides co-aggregated on ASC specks. Results are representative of two independent experiments.
(DOCX)

S2 Fig Co-aggregation of ovalbumin on ASC speck is not due to unspecific interactions between fluorescent labels. (A) cOVA-EYFP construct was expressed either in the absence (empty vector, pcDNA3) or in the presence of three inflammasome components (caspase-1, ASC, NLRP3) in HEK293T cells. As control, EYFP was expressed in the absence and presence of inflammasome components. (B) Western blotting analysis of samples in (A). Intensity of cOVA-EYFP band is much less compared to EYFP. Results are representative of two independent experiments.
(DOCX)

S3 Fig Engulfed ASC speck is slowly degraded. PMA differentiated stably EGFP-ASC expressing THP-1 macrophages were incubated with purified mCherry-tagged ASC specks. When THP-1 cells were imaged over 30 minutes without a growth chamber, cells underwent membrane blebbing. Under these conditions, tight phagolysosomal membrane around the engulfed ASC spec loosened. The space between phagolysosomal membrane and largely intact engulfed ASC speck was observed, which was filled with degradation products of ASC speck (mCherry signal). Fluorescence intensity of engulfed ASC speck was greater than free mCherry signal in the phagolysosomal space. However, mCherry signal was saturated to show both compartments in the same image.
(DOCX)

S1 Methods (DOCX)

S1 Table Peptide sequences used in EGFP-peptide constructs. (DOCX)
