# Supplementary material for: Artificial Loading of ASC Specks with Cytosolic Antigens
Source: PLoS One. 2015 Aug 10;10(8):e0134912. doi: 10.1371/journal.pone.0134912 (PMC4530869; doi:10.1371/journal.pone.0134912)
Supplement: S1 Fig — Three randomly chosen 26 amino acid long peptide encoding sequences from ampicillin resistance gene were cloned to C-terminus of EGFP. When co-expressed with mCherry-tagged ASC in HEK293T cells, only EGFP-peptide 1 co-aggregated on ASC speck, but EGFP-peptide 2 and-peptide 3 did not. EGFP-hydrophobic peptides but not EGFP-hydrophilic peptides co-aggregated on ASC specks. Results are representative of two independent experiments. (DOCX) [file pone.0134912.s001.docx]

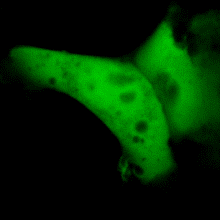

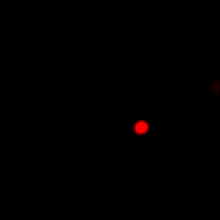

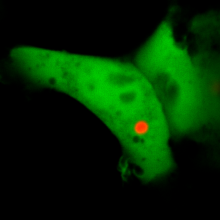


**EGFP-peptide 2**


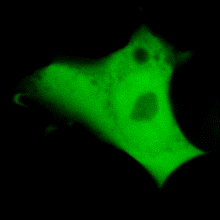

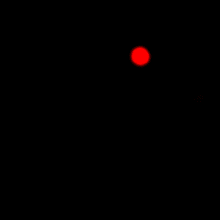

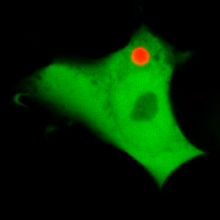


**EGFP-peptide 3**


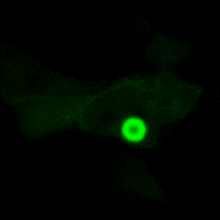

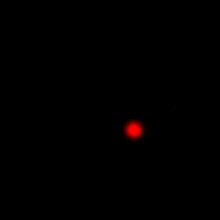

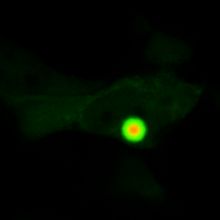


**EGFP-peptide 1**

**S1 Fig. | Co-aggregation of EGFP-peptide constructs on ASC specks.** Three randomly chosen 26 amino acid long peptide encoding sequences from ampicillin resistance gene were cloned to C-terminus of EGFP. When co-expressed with mCherry-tagged ASC in HEK293T cells, only EGFP-peptide 1 co-aggregated on ASC speck, but EGFP-peptide 2 and -peptide 3 did not. EGFP-hydrophobic peptides but not EGFP-hydrophilic peptides co-aggregated on ASC specks. Results are representative of two independent experiments.
