# Supplementary material for: Artificial Loading of ASC Specks with Cytosolic Antigens
Source: PLoS One. 2015 Aug 10;10(8):e0134912. doi: 10.1371/journal.pone.0134912 (PMC4530869; doi:10.1371/journal.pone.0134912)
Supplement: S2 Fig — (A) cOVA-EYFP construct was expressed either in the absence (empty vector, pcDNA3) or in the presence of three inflammasome components (caspase-1, ASC, NLRP3) in HEK293T cells. As control, EYFP was expressed in the absence and presence of inflammasome components. (B) Western blotting analysis of samples in (A). Intensity of cOVA-EYFP band is much less compared to EYFP. Results are representative of two independent experiments. (DOCX) [file pone.0134912.s002.docx]

**B**


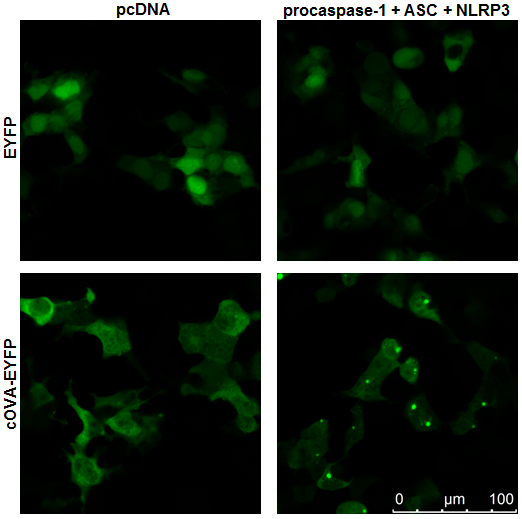

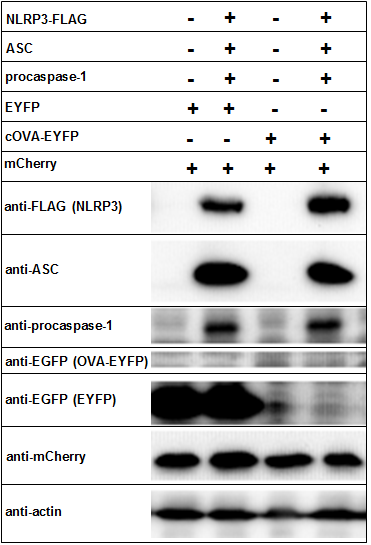


**A**

**S2 Fig. | Co-aggregation of ovalbumin on ASC speck is not due to unspecific interactions between fluorescent labels.** (A) cOVA-EYFP construct was expressed either in the absence (empty vector, pcDNA3) or in the presence of three inflammasome components (caspase-1, ASC, NLRP3) in HEK293T cells. As control, EYFP was expressed in the absence and presence of inflammasome components. (B) Western blotting analysis of samples in (A). Intensity of cOVA-EYFP band is much less compared to EYFP. Results are representative of two independent experiments.
