# Supplementary material for: Artificial Loading of ASC Specks with Cytosolic Antigens
Source: PLoS One. 2015 Aug 10;10(8):e0134912. doi: 10.1371/journal.pone.0134912 (PMC4530869; doi:10.1371/journal.pone.0134912)
Supplement: S3 Fig — When THP-1 cells were imaged over 30 minutes without a growth chamber, cells underwent membrane blebbing. Under these conditions, tight phagolysosomal membrane around the engulfed ASC spec loosened. The space between phagolysosomal membrane and largely intact engulfed ASC speck was observed, which was filled with degradation products of ASC speck (mCherry signal). Fluorescence intensity of engulfed ASC speck was greater than free mCherry signal in the phagolysosomal space. However, mCherry signal was saturated to show both compartments in the same image. (DOCX) [file pone.0134912.s003.docx]

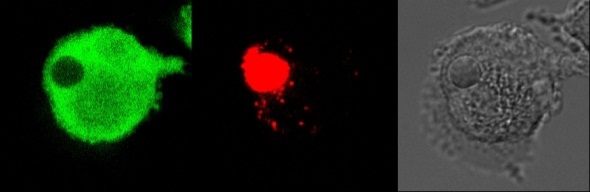

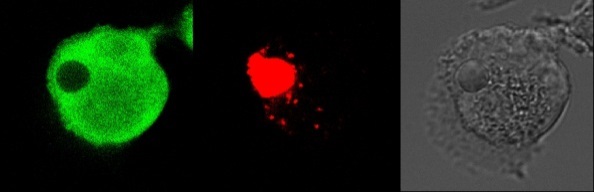

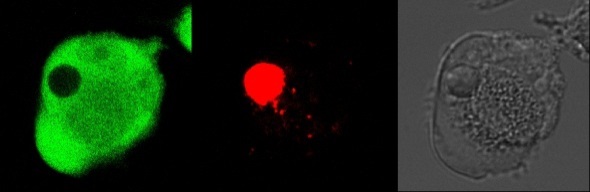

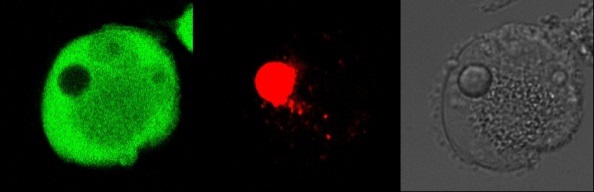

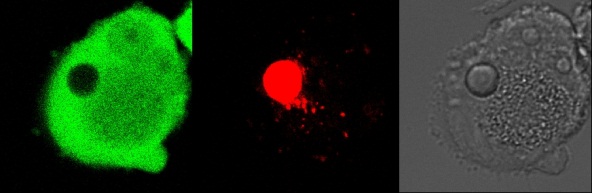

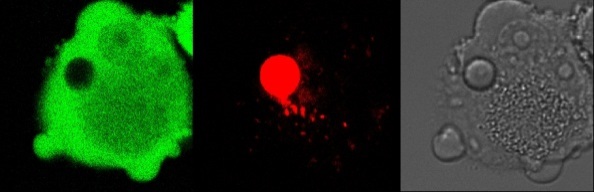

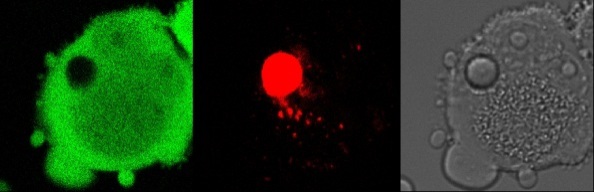

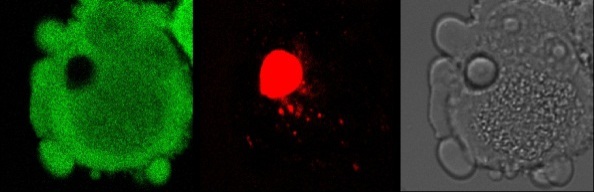

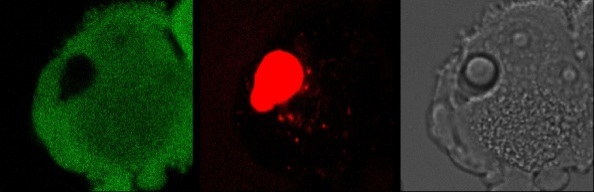

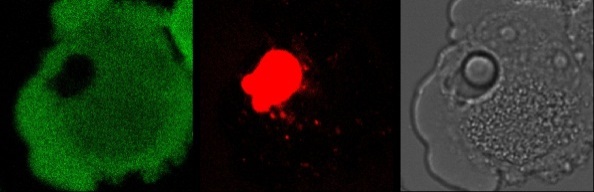

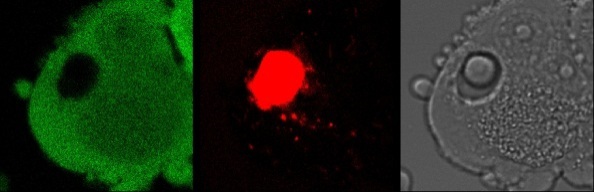

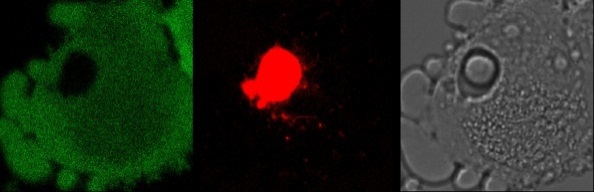


t=0

t=100

t=200

t=300

t=400

t=500

t=600

t=700

t=800

t=900

t=1000

t=1100

**Bright field**

**EGFP-ASC stable**

**engulfed ASC speck**

**Bright field**

**EGFP-ASC stable**

**engulfed ASC speck**

**S3 Fig. | Engulfed ASC speck is slowly degraded.** PMA differentiated stably EGFP-ASC expressing THP-1 macrophages were incubated with purified mCherry-tagged ASC specks. When THP-1 cells were imaged over 30 minutes without a growth chamber, cells underwent membrane blebbing. Under these conditions, tight phagolysosomal membrane around the engulfed ASC spec loosened. The space between phagolysosomal membrane and largely intact engulfed ASC speck was observed, which was filled with degradation products of ASC speck (mCherry signal). Fluorescence intensity of engulfed ASC speck was greater than free mCherry signal in the phagolysosomal space. However, mCherry signal was saturated to show both compartments in the same image.
