# Supplementary material for: Artificial Loading of ASC Specks with Cytosolic Antigens
Source: PLoS One. 2015 Aug 10;10(8):e0134912. doi: 10.1371/journal.pone.0134912 (PMC4530869; doi:10.1371/journal.pone.0134912)
Supplement: S1 Methods — (DOCX) [file pone.0134912.s004.docx]

**Sahillioğlu and Özören, 2015**

**Supplementary Methods**

**Plasmids.** pEGFP-hASC and pmCherry-hASC plasmids were cloned by subcloning human ASC cDNA derived from pcDNA3-hASC plasmid into pEGFP-C3 (Clontech, USA) and pmCherry-C3.1 (in-house produced) vectors between HindIII and EcoRI sites. pcOVA-EYFP plasmid was cloned by subcloning of cytoplasmic ovalbumin cDNA (1-48aa secretion signal deleted) from pCI-neo-sOVA plasmid (Addgene plasmid 25098, kindly provided by Prof. Pedro Lowenstein, Cedars-Sinai Medical Center) into pEYFP-N1 vector (Clontech, USA) between NheI-BglII sites. Ubiquitin B cDNA was subcloned from pCGN-HA-Ubiquitin plasmid into pmCherry-C3.1 to obtain pmCherry-UBB. DNA sequences encoding short peptides were cloned between BglII and EcoRI sites of pEGFP-C3 vector (Supplementary Table S2). pEGFP alone and pmCherry alone plasmids have the same vector backbone as pEGFP-C3 except they lack the multiple cloning site. EGFP-ASC encoding DNA from pEGFP-hASC and mCherry-ASC encoding DNA from pmCherry-hASC were subcloned into pLenti-Ef1a vector to yield lentiviral plasmids pLenti-Ef1a-EGFP-ASC and pLenti-Ef1a-mCherry-ASC. These constructs were used together with packaging plasmids pCMVdeltaR8.74 and pMD2.G plasmids for virus production. pCMVdeltaR8.74 and pMD2.G were kindly provided by Prof. Karl Deisseroth (Stanford University). pcDNA3-NLRP3-FLAG, pcDNA3-hASC, pcDNA3-procaspase-1-FLAG, pCGN-HA-Ubiquitin plasmids were kindly provided by Prof. Gabriel Nunez (University of Michigan).

**Purification of ASC specks.** HEK293T cells were transfected with ASC encoding plasmids by calcium phosphate method. 24 h after transfection, cells were lysed by sonication. ASC specks were sepearated from soluble proteins and enriched by repeated low speed centrifugation cycles at 200 g.

**Antibodies.** Anti-FLAG (2368S, CST, USA), anti-Caspase-1 (sc-515, Santa Cruz), anti-EGFP (Vatoz, Turkey), anti-ASC (kindly provided by Dr Masumoto, Jichi Medical University), anti-mCherry (kindly provided by Dr Arzu Celik, Bogazici University) were used for Western blotting.

**Cell culture.** HEK293(F)T cells were kindly provided by Prof. Maria Soengas (CNIO) and maintained in DMEM containing 10% FBS. THP-1 cells were kindly provied by Prof. Ahmet Gül (Istanbul University) and maintained in RPMI containing 10% FBS.
