# Supplementary material for: Artificial Loading of ASC Specks with Cytosolic Antigens
Source: PLoS One. 2015 Aug 10;10(8):e0134912. doi: 10.1371/journal.pone.0134912 (PMC4530869; doi:10.1371/journal.pone.0134912)
Supplement: S1 Table — (DOCX) [file pone.0134912.s005.docx]

**S1 Table | Peptide sequences used in EGFP-peptide constructs.**

| **Name** | **Length** | **Sequence** | **Co-aggregation on ASC speck** |
| --- | --- | --- | --- |
| Peptide 1 | 26 | YSDLMSIQHFRVALIPFFAAFCLPVF | + |
| Peptide 2 | 26 | YSDLAHPETLVKVKDAEDQLGARVGY | - |
| Peptide 3 | 26 | YSDLIELDLNSGKILESFRPEERFPM | - |
| C3 | 26 | YSDLELKLRILQSTVPRARDPPDLDN | + |
| Peptide 1_19aa | 19 | YSDLVALIPFFAAFCLPVF | + |
| Peptide 1_12aa | 12 | YSDLAAFCLPVF | - |
| Peptide 1_8aa | 8 | YSDLLPVF | - |
| C3_19aa | 19 | YSDLQSTVPRARDPPDLDN | - |
| hydrophobic 1 | 19 | YSDLIVLLVFFLFVLFIII | + |
| hydrophobic 2 | 19 | YSDLLFILFVIVIVFIFLL | + |
| hydrophilic 1 | 19 | YSDLKNNERKDKDDENRER | - |
| hydrophilic 2 | 19 | YSDLRDNNRKDKEEEEDNR | - |
